# Transforming medical device clinical trials with agentic AI: a Taiwan-specific framework integrating smart data, regulatory evidence, and trial optimization

**DOI:** 10.3389/fdgth.2026.1934685

**Published:** 2026-10-30

**Authors:** Wo Yun Lee, Chien-Chen Lin, Guang-Huar Young, Robert Y. L. Wang

**Affiliations:** 1 Biotechnology Industry Master and Ph.D. Program, Chang Gung University, Taoyuan, Taiwan; 2 Blue Blood Biotech Corp., Taipei, Taiwan; 3 Sanar Biotech Corp., Taipei, Taiwan; 4 Department of Biomedical Sciences, College of Medicine, Chang Gung University, Taoyuan, Taiwan; 5 Department of Otolaryngology–Head and Neck Surgery, Chang Gung Memorial Hospital, Keelung, Taiwan

**Keywords:** adaptive trial design, agentic AI, medical device clinical trial, real-world evidence, regulatory science, software as a medical device

## Abstract

Medical device clinical trials present distinctive regulatory and methodological challenges—including device-specific classification, engineering-based endpoints, operator-dependent performance, device-related safety considerations, and lifecycle evidence obligations—that differ from those of pharmaceutical trials. In our structured synthesis, we identified no published framework that explicitly integrates agentic AI into medical device clinical-trial workflows within Taiwan's regulatory context. Drawing on a structured synthesis of 39 core sources (29 scholarly publications and 10 regulatory or standards documents), supplemented by targeted contextual literature on AI governance and implementation, we developed a five-layer framework comprising Regulatory Decision, Evidence Requirement, Data Integration, Agentic AI Optimization, and Adaptive Feedback. The framework links bounded agentic AI functions to TFDA regulatory decision-making, device-specific evidence requirements, device-derived and real-world data, and lifecycle evidence generation. Cross-cutting governance considerations include algorithmic bias and representativeness, transparency and traceability, human oversight and accountability, cybersecurity and privacy, and model-drift and lifecycle change management. The framework is a contextual proposal for Taiwan rather than a validated or implemented system. We provide actionable recommendations for regulators, investigators, AI developers, and infrastructure planners. External assessment and prospective feasibility testing are required before implementation.

## 1 Introduction

Medical device clinical trials face rising evidentiary expectations, increasingly complex device–user interactions, and tighter regulatory constraints on clinical data generation (1, 2). Unlike drug trials, device trials involve engineering performance specifications, usability and operator learning curves, device-related adverse events, and post-market performance monitoring. These characteristics create opportunities for data-driven trial optimization.

Prior work has examined agentic AI across several trial functions: protocol optimization (ClinicalReTrial, TrialGenie) (1, 2); multi-agent reasoning (Clinical Agent) (3); LLM-based recruitment (PRISM) (4); risk-based monitoring validated in pharmaceutical quality-management contexts (5); and foundation models spanning multiple trial tasks (Panacea) (6). Collectively, these studies illustrate the potential of agentic or agent-like AI systems to support specific trial functions. However, none of these systems was designed for, or validated against, medical device trial requirements, which differ in engineering endpoints, operator-dependent learning curves, usability obligations, device-specific adverse-event classification and lifecycle evidence generation, including post-market clinical follow-up where applicable. No existing framework addresses agentic AI within Taiwan's TFDA regulatory environment.

We distinguish three AI paradigms: conventional AI/ML, which primarily performs predefined prediction, classification, or optimization tasks; generative AI, which produces content in response to prompts; and agentic AI, which we define as a system architecture that pairs a reasoning model with tools, persistent state, goal-directed planning, feedback evaluation, and human control mechanisms (1, 2, 6, 7). In our framework, agentic AI does not autonomously control a trial. It handles circumscribed tasks—evidence retrieval, eligibility screening, protocol-adherence monitoring, endpoint review, and draft documentation—while consequential decisions stay with the investigator. Taiwan offers mature hospital information systems, the National Health Insurance Research Database (NHIRD), and a growing device-innovation ecosystem, but a practical framework linking AI-driven trial optimization with TFDA regulatory decision-making and real-world evidence generation is lacking. Although the two illustrative cases focus on orthopaedic smart-implant data, the framework is intended to be adaptable across device categories, although its applicability beyond orthopaedic examples remains to be evaluated.

The practical urgency of this framework is underscored by Taiwan's growing AI-in-healthcare ecosystem. The TFDA's Smart Medical Device Information and Matchmaking Platform (AIMD) provides regulatory consultation, classification guidance, and a cumulative list of approved AI/ML medical devices. Workshops on SaMD validation and lifecycle management, co-organized by TFDA and industry bodies, reflect increasing regulatory attention to the intersection of AI and medical device governance. At the same time, innovative AI-assisted regulatory tools—such as systems that combine large language models with retrieval-augmented generation for device classification and pre-submission analysis—are beginning to automate portions of the regulatory intelligence workflow. These developments create both the opportunity and the need for a structured framework that situates agentic AI within the full device clinical trial lifecycle, from regulatory pathway determination through post-market evidence generation.

This paper responds to that need by proposing a five-layer framework—Regulatory Decision, Evidence Requirement, Data Integration, Agentic AI Optimization, and Adaptive Feedback—designed to integrate bounded agentic AI functions into the TFDA-regulated device trial lifecycle. Unlike previous AI-in-clinical-trials frameworks, which focus predominantly on pharmaceutical applications, the proposed architecture explicitly addresses device-specific requirements: engineering-based endpoints, operator-dependent performance assessment, device-related adverse-event classification, SaMD regulatory-status determination, and lifecycle-long post-market clinical follow-up obligations. Two illustrative cases—one drawn from orthopaedic smart-implant clinical data, the other from a comparative regulatory pathway analysis—demonstrate how the framework layers operate under different evidentiary conditions, though neither case constitutes validation of the framework itself.

## 2 Methodology and evidence synthesis

### 2.1 Study design

We developed the framework through four steps: structured literature synthesis, functional capability mapping, primary regulatory document analysis, and illustrative case-based specification. This is a conceptual framework study, not a systematic review or primary empirical investigation. The two illustrative cases are secondary applications from published literature; they clarify potential use and do not constitute validation or original clinical research.

### 2.2 Literature search and eligibility

A structured search was conducted across PubMed, IEEE Xplore, arXiv, medRxiv, and official repositories of the FDA, TFDA, European Commission, ISO, and IMDRF. The January 2020–July 2026 date range was applied to scholarly literature; primary legislation, regulatory guidance, and standards documents were selected according to current relevance and applicability and were not restricted by publication date. The first focused on agentic AI and clinical-trial applications: (“agentic AI” OR “autonomous AI” OR “multi-agent system” OR “LLM agent”) AND (“clinical trial” OR “medical device” OR “regulatory” OR “trial optimization”). The second targeted smart-device and orthopaedic evidence: (“smart implant” OR “wearable sensor”) AND (“knee arthroplasty” OR “total knee replacement”) AND (“clinical outcomes” OR “patient-reported outcomes” OR “gait metrics”). Eligible sources addressed agentic, autonomous, or multi-agent AI applied to clinical-trial functions; sensor-derived or device-generated outcomes in orthopaedic or smart-device settings; or relevant regulatory and standards requirements. Of the 247 records initially identified, 158 were excluded before formal title and abstract screening: 35 were duplicates and 123 were judged clearly outside the prespecified search domains during an initial relevance assessment. The remaining 89 records underwent formal title and abstract screening, after which 50 were excluded. A total of 39 core sources were retained for the structured synthesis, comprising 29 scholarly publications and 10 regulatory or standards documents. This synthesis was designed to support conceptual framework development rather than to constitute a systematic or scoping review; therefore, PRISMA-ScR procedures were not applied. Seven additional targeted references were subsequently consulted to contextualize the five governance domains discussed in Section 4; these references were not included in the 39-source structured-synthesis count. One further source is cited to document a Taiwan-specific regulatory-AI tool.

### 2.3 Framework construction

We first mapped AI capabilities against documented device-trial pain points under ISO 14155:2026 and TFDA requirements, then derived the five-layer architecture from the intersection of AI functions and device-trial lifecycle stages, and finally developed two illustrative cases. Iterative discussion among the authors refined the architecture. We did not convene a formal Delphi panel or external expert group, a limitation we address in Section 4.4.

### 2.4 TFDA regulatory framework analysis

TFDA legislative documents and guidance were obtained from official TFDA and Ministry of Health and Welfare sources (8, 9). Taiwan's Medical Devices Act (2021) establishes three-tier classification (Class I–III), with Class II and III devices generally requiring Clinical Trial Permits (CTPs), except for investigations determined to involve no significant risk under applicable TFDA requirements (9). Medical device clinical investigations in Taiwan are governed by the TFDA Regulations on Good Clinical Practice for Medical Devices (10). ISO 14155:2026 provides the current international device-specific GCP standard (11), while ICH E6(R3) offers complementary cross-sector principles for trial quality and risk management (12). The 2022 SaMD guidance establishes risk-based classification for AI- and algorithm-driven software (13). Post-market surveillance and adverse-event reporting obligations apply under Taiwan medical-device regulations; EU MDR PMCF (Annex XIV Part B) is presented in this framework as an international comparator rather than a legally equivalent Taiwan requirement (14, 15). A detailed regulatory comparison is presented in Table 2; these systems share risk-proportionality principles but differ in classification criteria and legal scope and are not treated as legally equivalent.

## 3 Results: policy and regulatory assessment

### 3.1 Literature mapping

The synthesis retained 39 sources: 13 AI-related trial-workflow, agent-evaluation, or governance publications; 16 orthopaedic or smart-device clinical publications; and 10 regulatory or standards documents. AI-related sources span protocol optimization and redesign (1, 2), interpretable prediction and statistical-programming automation (7, 16), multi-agent reasoning (3), real-time quality monitoring (5), clinical AI-agent evaluation (17), trial search and eligibility matching (4, 6), and governance or integrative reviews (18–21). To date, none has been designed for—or validated through—a TFDA device-trial submission. Table 1 summarizes selected sources.

**Table 1:** Literature mapping of selected representative sources from the 39-source core synthesis. The January 2020–July 2026 search period applied to scholarly literature only; regulatory and standards documents were included by current applicability. Additional governance and Taiwan-specific contextual sources were consulted separately and are excluded from the 39-source count.

Ref	Study	Domain	Core contribution	Relevance to device trials
1	Li et al. 2026 (TrialGenie)	RWD-enabled trial design	Five-agent framework; emulation+RWE feasibility	RWE-informed device trial design
2	Xing et al. 2026 (ClinicalReTrial)	Protocol optimization	Self-evolving AI agent for iterative protocol redesign	Adaptive protocol refinement in device trials
3	Yue et al. 2024 (ClinicalAgent)	Multi-agent reasoning	ReAct-based planning with knowledge retrieval	Multi-agent coordination for complex device workflows
4	Gupta et al. 2024 (PRISM)	Recruitment/eligibility	LLM-based semantic EHR matching	Device patient screening and recruitment support
5	Jiang et al. 2025	Real-time monitoring	Digital risk-based quality management (RBQM)	Protocol-adherence and safety signal monitoring
6	Lin J et al. 2024 (Panacea)	Foundation model	Medical foundation model spanning trial tasks	Multi-function AI backbone for trial workflows
7	Liu et al. 2025 (AutoCT)	Interpretable prediction	Automating clinical trial prediction with LLM agents	Statistical automation for device endpoints
16	Yan 2026 (ClinAgent)	Statistical programming	Five-layer architecture for autonomous stat programming	Automated statistical documentation generation
22	Guild et al. 2024	Smart implant gait	Gait vs. PROMs; adjusted rho 0.31–0.41 in TKA	Dual-endpoint design evidence for sensor trials
23	Wu KA et al. 2025	PROMs correlation	Prospective PROM–gait study; Pearson r ≈ 0.2–0.3	Sensor-endpoint validation for orthopaedic trials
24	Lee et al. 2026	AI device regulation	70 FDA-cleared orthopaedic AI devices; 22.8% untested	Evidence-gap context for TFDA AI device pathways
25	Hoogervorst et al. 2023	Registry PMCF	EU cardiovascular/orthopaedic registry quality review	Registry-based PMCF automation target

Reference numbers correspond to the renumbered reference list. Only representative sources are shown.

**Table 2:** Comparison of TFDA, FDA, and EU MDR regulatory approaches to AI-enabled medical devices, with relevance to the proposed five-layer framework. Legal equivalence between jurisdictions is not implied.

Dimension	TFDA (Taiwan)	FDA (USA)	EU MDR	Framework layer
Device classification	Class I–III; risk-based classification	Class I–III; risk-based classification	Class I, IIa, IIb, III; MDR Annex VIII	Layer 1: Regulatory Decision
SaMD/AI guidance	2022 medical-software classification guidance; AIMD support	AI-enabled device guidance; TPLC approach; final PCCP guidance	MDR+AI Act where applicable; MDCG 2019-11	Layers 1 & 4
Clinical investigation pathway	Prior TFDA approval generally required; announced non-significant-risk investigations may be exempt	IDE requirements depend on significant-risk status; abbreviated requirements for NSR studies	Clinical investigations under MDR Article 62 and Annex XV	Layer 2: Evidence Requirement
GCP/clinical-investigation standard	TFDA GCP for Medical Devices; ISO 14155-informed	21 CFR Part 812 and applicable FDA GCP requirements	MDR clinical-investigation requirements; ISO 14155 used as relevant standard	Layers 2 & 4
Post-market evidence	PMS and adverse-event reporting; no EU-equivalent PMCF requirement	Post-approval studies, 522 surveillance, and RWE mechanisms	PMCF under MDR Annex XIV Part B; PSUR where applicable	Layer 5: Adaptive Feedback
Data privacy	PDPA: specified-purpose and necessary-scope principles, security safeguards, and applicable international-transfer conditions	HIPAA and applicable electronic-record/privacy requirements	GDPR and applicable AI Act data-governance requirements	Layers 3 & 4
AI lifecycle change management	PCCP guidance issued for AI/ML-enabled medical device software; applicability to trial-support agentic AI remains unclear	Final PCCP guidance and TPLC-based lifecycle management	Substantial-modification controls and ongoing monitoring under applicable MDR/AI Act requirements	Layers 4 & 5

From an international perspective, the FDA reported by 2025 that more than 1,000 AI-enabled medical devices had been authorized through established premarket pathways. The European Union's AI Act entered into force in 2024, with its provisions becoming applicable in phases, and introduces risk-based obligations that may apply to AI systems incorporated into or functioning as medical devices alongside applicable MDR requirements. The IMDRF SaMD Working Group's Clinical Evaluation guidance (N41:2017) remains a foundational international reference for SaMD clinical evaluation, organizing the evidence framework around valid clinical association, analytical validation, and clinical validation. Against this international landscape, a Taiwan-specific framework should consider areas of regulatory convergence while preserving jurisdiction-specific requirements, particularly TFDA device classification and clinical-investigation approval pathways and PDPA-governed data processing.

Taiwan's regulatory environment for AI-enabled medical devices has matured considerably since the Medical Devices Act came into effect in 2021. TFDA published its Reference Guidance for the Classification of Medical Software in 2022, establishing a risk-based classification framework for SaMD that considers the seriousness of the health condition addressed, the significance of the information provided to clinical decision-making, and whether the software is intended to diagnose, treat, or inform. As of mid-2026, TFDA has published a cumulative list of AI/ML-based medical devices approved in Taiwan, covering applications in radiology, pathology, ophthalmology, and cardiology. However, there remains no AI/ML device approved by TFDA specifically for clinical trial workflow functions such as protocol optimization, eligibility screening, or adaptive monitoring—the functions addressed by this framework. Meanwhile, emerging AI-assisted regulatory tools such as MEDPilot, developed by Taiwan's National Applied Research Laboratories, demonstrate how large language models combined with retrieval-augmented generation can accelerate pre-submission preparation for device classification, predicate identification, and substantial-equivalence analysis (22). Such tools illustrate a broader trend:AI is entering not only clinical decision-making but also the regulatory intelligence and submission-preparation layers of the device lifecycle, reinforcing the relevance of the proposed framework's Regulatory Decision Layer.

### 3.2 Proposed framework

The framework integrates regulatory decision-making, evidence requirements, data integration, and agentic AI-supported trial optimization. Regulatory classification and pathway shape the applicable evidentiary requirements; the data layer provides conventional clinical outcomes and smart-device signals; the Agentic AI Layer supports bounded functions such as eligibility assessment, endpoint review, recruitment planning, monitoring, and evidence synthesis; and the adaptive layer closes the loop between trial data, redesign, and regulatory feedback. We place agentic AI in a discrete layer—not because AI activity is limited to that layer, but for three practical reasons: it creates a clear boundary for human oversight and regulatory review; it keeps the AI components modular, so they can be upgraded or disabled independently; and it facilitates independent assessment of the AI component's potential SaMD status and associated lifecycle controls. It has bidirectional influence on the Regulatory Decision, Data Integration, and Adaptive Feedback Layers (Figure 1a). Figure 1b provides an orthopaedic smart-implant instantiation.

**Figure 1:**
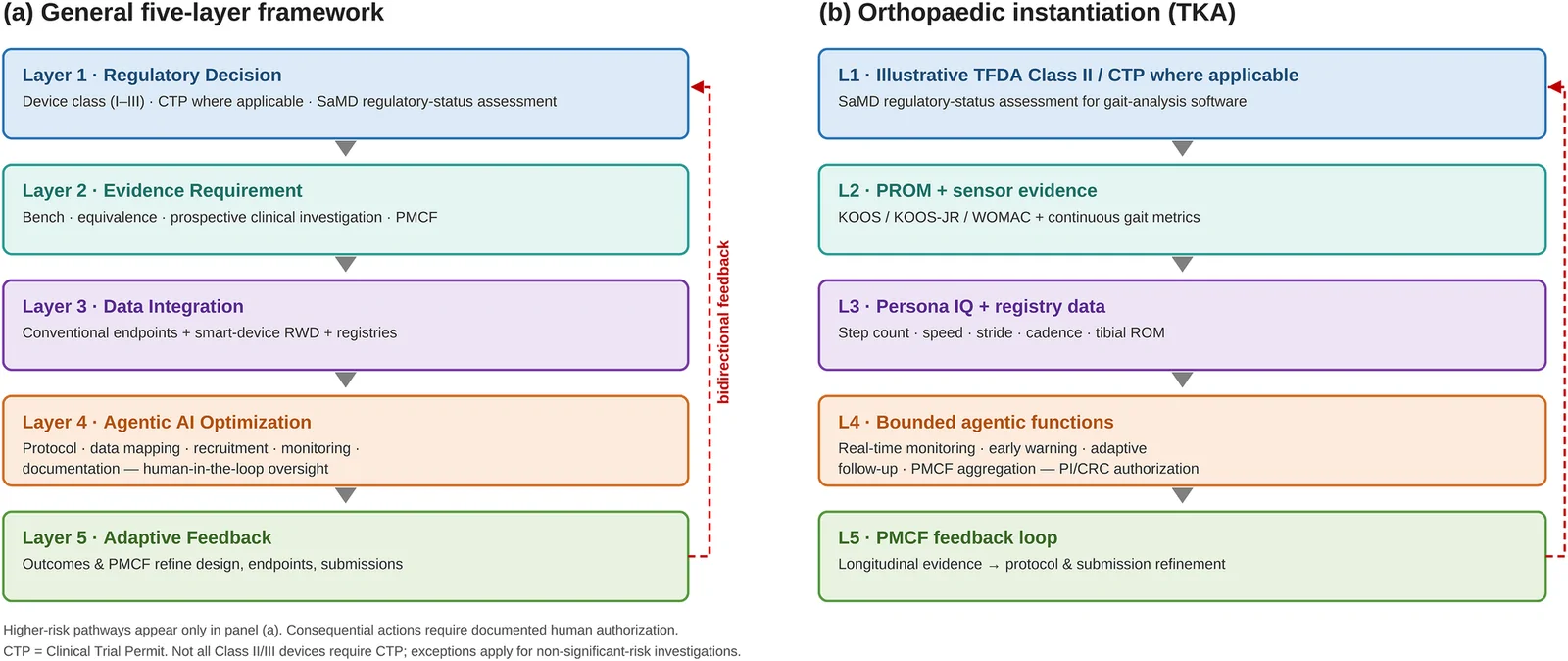
Five-layer agentic AI framework for medical device clinical trials. **(a)** General framework showing the Regulatory Decision, Evidence Requirement, Data Integration, Agentic AI Optimization, and Adaptive Feedback Layers. The Agentic AI Layer is presented as a discrete layer for auditability and modularity, while interacting bidirectionally with the regulatory, data, and feedback layers. All layers operate under applicable regulatory, clinical-investigation, and data-governance requirements. **(b)** Orthopaedic illustrative instantiation in total knee arthroplasty using a smart tibial implant, showing an illustrative TFDA Class II/CTP context, PROM-plus-sensor evidence, integration of conventional and continuous gait data, bounded agentic AI functions, and PMCF feedback. Higher-risk pathways (e.g., FDA PMA-equivalent) are shown only in panel **(a)** Human authorization by the PI and/or CRC is retained for consequential actions.

The IMDRF SaMD Clinical Evaluation framework offers useful parallels for structuring AI governance within the proposed architecture. IMDRF distinguishes three evaluation levels: valid clinical association (whether the AI output is associated with a targeted clinical condition), analytical validation (whether the AI achieves its technical specifications), and clinical validation (whether the AI achieves its intended purpose for a target population). In our framework, valid clinical association maps primarily to Layers 1–2, where the clinical relevance of agentic functions is assessed; analytical validation maps to Layer 4, where individual agent performance is tested; and clinical validation maps to the interaction of Layers 4 and 5, where agentic outputs are evaluated in the context of actual trial conduct. This alignment is offered as a structural parallel for organizing evaluation, not an assertion that IMDRF SaMD evaluation applies directly to trial support AI.

### 3.3 Case 1: smart implant data as a trial optimization input

Neither case involves new participants or original data; both draw entirely on published results. In TKA, sensor-enabled tibial implants (e.g., Zimmer Biomet Persona IQ) continuously capture step count, walking speed, stride length, cadence, and tibial range of motion (23, 24), complementing PROMs such as KOOS, KOOS-JR, and WOMAC collected at discrete time points (25, 26).

Sensor-derived gait measures and PROMs capture related but non-interchangeable recovery dimensions. Guild et al. (2024) retrospectively evaluated 88 TKA patients and found gait improvement over 12 weeks but weak associations with PROMs (absolute Spearman's rho in the 0.31–0.41 range for selected adjusted pairs) (27); Wu et al. prospectively evaluated 152 patients and reported consistently weak PROM–gait associations with stronger internal consistency for objective measures (28). These findings point toward dual-endpoint designs as a direction worth testing prospectively, though clinically meaningful thresholds and standardized validation are still needed before any regulatory application (26, 27). Continuous monitoring may also aid early warning: Dundon et al. detected deterioration 4.5 weeks post-TKA before a scheduled visit, prompting earlier manipulation under anaesthesia (29), and cohort reviews suggest gait trajectories offer early-recovery benchmarks while noting limited prospective evidence (30). Illustrative studies further show the heterogeneity of data streams that a future agentic workflow might encounter. Park et al. and Gimm et al. used machine-learning models to predict failure to achieve clinically important postoperative improvement after TKA (31, 32), while Sharma et al. reviewed AI approaches for predicting postoperative orthopaedic complications (33). Gould et al. combined administrative and registry data to model 30-day readmission after TKA (34). Jung et al. evaluated wearable motion-tracker–supported home rehabilitation in a randomized trial, and King et al. reviewed wearable-sensor applications for remote rehabilitation following knee arthroplasty (35, 36). These studies are included as illustrative examples of heterogeneous clinical, registry, and wearable data sources that could inform future AI-enabled workflows; they do not validate the proposed framework.

Within the framework, these streams could support five bounded functions (2): real-time monitoring of device-derived endpoints against benchmarks [Layer 4→5] (7); early-warning identification of delayed recovery [Layer 4→5, feedback to Layer 3] (1); adaptive follow-up on validated deterioration signals [Layer 4→5] (3); multi-site aggregation of post-market clinical evidence for regulatory evidence [Layer 4→5→1]; and (5) longitudinal synthesis informing protocol and endpoint design [Layer 5→Layers 1,2]. Each function is decision support; the investigator retains authority over consequential actions. Implementation in Taiwan requires PDPA-compliant governance, IRB approval, and data-access arrangements; UDI–record linkage could strengthen PMCF, and deployment across sites should not assume equivalent AI performance without site-specific assessment.

### 3.4 Case 2: regulatory pathway comparison

Case 2 is a comparative regulatory-document analysis. AI's role changes with evidentiary burden. Under the FDA 510(k) pathway, AI could support predicate identification, substantial-equivalence mapping, and evidence-gap analysis; certain TFDA Class II applications permit comparison with a legally marketed similar device, creating a limited procedural resemblance without equivalent legal criteria or evidentiary requirements. For higher-evidence pathways (FDA PMA; higher-risk TFDA contexts requiring prospective evidence), AI could support protocol optimization, eligibility refinement, endpoint selection, recruitment, monitoring, and documentation.

The regulatory landscape underscores the gap: among 70 FDA-cleared orthopaedic AI/ML devices, clearances rose from 3.0/year (2017–2019) to 16.6/year (2022–2024), yet 22.8% underwent no clinical testing and only 8.6% were validated by prospective trial (37). Registry evidence is likewise uneven—European arthroplasty registries can contribute EU MDR PMCF data but vary in quality and often lack comparative PROM data for high-risk implants (38). Systematic registry-based aggregation could narrow these gaps, though it does not by itself justify autonomous AI reporting or define TFDA-specific PMCF requirements. For lower-evidence activities, AI mainly supports regulatory intelligence [Layer 4 on Layer 1] and evidence mapping [Layer 4 on Layer 2]; for higher-evidence pathways, emphasis shifts to protocol/endpoint optimization [Layer 4 on Layer 2], eligibility support [Layer 4 on Layer 3], adaptive monitoring [Layers 4,5], and longitudinal aggregation [Layer 5→Layers 1,2]. All mappings remain jurisdiction-dependent and await empirical testing.

This regulatory-pathway comparison has direct policy implications for Taiwan. As TFDA continues to develop its AI/ML medical device ecosystem—including the Smart Medical Device Information and Matchmaking Platform (AIMD), dedicated SaMD workshops, and regulatory consultation services—the framework's layered approach offers a structured way to think about where AI adds value at different points in the regulatory pathway. For lower-risk devices proceeding through comparison-based pathways, AI can accelerate the regulatory intelligence phase without requiring extensive prospective clinical data. For higher-risk devices requiring CTPs and prospective evidence, AI's role expands into the trial-conduct and monitoring phases, where the governance requirements of Section 4 become most critical. This graduated engagement model aligns with the risk-proportionality principle that underpins both TFDA's classification system and international frameworks such as the IMDRF's SaMD risk categorization matrix.

Three policy gaps emerge from the cross-jurisdictional comparison in Table 2. First, TFDA has established medical-device classification, clinical-investigation GCP, and medical-software guidance, but the regulatory status and lifecycle controls for agentic AI used primarily as trial-support infrastructure remain insufficiently specified. Second, compared with the FDA and EU approaches summarized above, the applicability of Taiwan's lifecycle change-control mechanisms to non-device trial-support agents remains unclear. Third, Taiwan has not yet articulated device-trial-specific expectations for incorporating AI-generated workflow outputs into clinical-investigation documentation, including requirements for human authorization, auditability, and cross-site performance assessment. These gaps do not imply an absence of regulation; rather, they identify areas in which existing TFDA requirements may require interpretation or supplementary guidance.

## 4 Governance assessment

Governance must address both the AI system's regulatory status and operational safeguards. AI performing medical-purpose functions or materially influencing clinical decisions may require independent assessment of whether it constitutes SaMD and what premarket or lifecycle controls apply (13, 39). Scoping evidence shows agent capability outpacing translational validation and governance (20), with persistent gaps in accountability, explainability, and oversight (21). The five governance domains outlined below respond to documented failures, not theoretical risks.

Taiwan's evolving regulatory infrastructure for AI-enabled medical devices provides the starting conditions for governance but does not yet offer device-trial-specific AI governance guidance. The TFDA's 2022 SaMD classification guidance addresses premarket requirements for diagnostic and therapeutic AI software, but the scenario in which AI operates as an agentic system within a clinical investigation—performing protocol-adherence monitoring, eligibility screening, or evidence synthesis—raises governance questions that fall between existing SaMD regulation and GCP requirements. Whether such systems require independent SaMD premarket assessment, and what lifecycle controls apply during an investigation, are questions that TFDA has not yet formally addressed. The governance domains outlined below are therefore proposed both as operational safeguards for investigators and as topics for future TFDA guidance development. Each domain is grounded in a documented international case that illustrates the real-world consequences of governance failure.

Taiwan's position at the intersection of several regulatory traditions—its Medical Devices Act draws on both FDA and EU MDR concepts, its data governance is anchored in the PDPA rather than GDPR or HIPAA, and its clinical investigation requirements reference ISO 14155 and ICH E6(R3)—creates a unique governance context. The five domains proposed here are not imported wholesale from any single jurisdiction; rather, they synthesize lessons from documented failures across multiple regulatory environments and translate them into operational requirements appropriate for TFDA-regulated device trials. Each domain is accompanied by a specific international case study that illustrates the consequences of governance failure, followed by Taiwan-specific mitigation measures that account for the PDPA, TFDA classification framework, and the practical realities of Taiwan's clinical investigation infrastructure.

### 4.1 Algorithmic bias and representativeness

Agentic AI trained on non-representative data may exhibit clinically important performance disparities across patient subgroups. Obermeyer et al. demonstrated that a widely used health-risk algorithm systematically underestimated the needs of Black patients because healthcare cost was used as a proxy for illness severity; correcting this bias increased the proportion of Black patients identified for additional care from 17.7% to 46.5% (40). Similarly, Kaur et al. identified subgroup-specific disparities in an ECG-based heart-failure prediction model, particularly among younger Black patients, despite acceptable overall performance; conventional mitigation approaches such as dataset rebalancing and race-stratified models did not fully resolve these disparities (41). These findings illustrate why aggregate model performance alone is insufficient for agentic AI used in medical device trials. Bias in eligibility screening could distort participant selection and trial representativeness, while differential performance in outcome prediction or recruitment could affect risk stratification and amplify existing disparities. For Taiwan-based device trials, mitigation should therefore include locally relevant validation datasets, pre-specified subgroup performance criteria, disaggregated performance reporting, and prospective monitoring throughout the trial, with predefined corrective actions when subgroup thresholds are not met. In Taiwan, the relevant fairness assessment should focus on locally salient demographic, clinical, and site-level subgroups rather than assuming that disparity patterns observed in other jurisdictions will reproduce unchanged.

### 4.2 Transparency and traceability

Two analyzes of FDA-cleared AI/ML medical devices document that transparency requirements are systematically unmet in current practice. A scoping review of 692 FDA-approved AI/ML devices found that only 1.9% had a linked peer-reviewed publication, 46.1% reported detailed performance results, and race/ethnicity data were available for just 3.6% (42). A concurrent conceptual analysis of clinical AI decision support systems found that outputs may lack sufficiently visible evidentiary provenance and an auditable basis for decision-making (43). In a trial context, an eligibility determination that cannot be traced to the applicable CIP version, the patient data accessed, the criterion operationalized, and the system's confidence level constitutes an undocumented deviation under ISO 14155:2026—a failure discovered not at output generation but at audit or regulatory inspection, by which point reconstruction may be impossible. Agentic AI systems must therefore maintain structured records of evidence sources, input–output provenance, tool-call logs, decision rationale, uncertainty indicators, and version history, supporting both real-time oversight and post-trial auditability. For Taiwan, traceability should also preserve the applicable TFDA guidance version, CIP version, data source, model or agent version, and human-authorization record so that decisions can be reconstructed for sponsor oversight, IRB review, audit, or regulatory inspection.

### 4.3 Accountability and human oversight

Deploying agentic AI in TFDA-regulated device trials does not transfer responsibility from the principal investigator or sponsor to the AI system; ISO 14155:2026 accountability obligations remain intact regardless of AI involvement. A 2024 regulatory case illustrates what accountability failure looks like in practice: Pieces Technologies, a clinical AI company operating in at least four US hospital systems, settled with the Texas Attorney General after an investigation found that the company's marketed hallucination-rate metrics for its AI-generated clinical summaries were likely inaccurate and may have deceived hospitals about product accuracy and safety (44). Physicians and nurses had been relying on outputs whose error rate had not been independently verified. The case established that AI systems used for clinical documentation are subject to consumer protection enforcement even in the absence of AI-specific regulation. In trial operations, three categories of consequential action must require documented human authorization before execution—protocol amendments, participant withdrawal, and safety escalation—with a timestamped record of who authorized the decision and on what basis. In Taiwan, these responsibilities should be mapped to the respective duties of sponsors, investigators, and authorized trial personnel under applicable TFDA GCP and institutional procedures rather than transferred to the AI system.

### 4.4 Cybersecurity and privacy

The breadth of data an agentic AI trial system may access—EHR, eCRF, laboratory results, wearable sensor streams, and regulatory documents simultaneously—creates a correspondingly broad exposure surface. The February 2024 Change Healthcare ransomware attack, which exploited a legacy remote-access portal lacking multifactor authentication, illustrates the consequences of inadequate foundational access controls in healthcare data systems (45). Attackers moved laterally for nine days undetected, and the root cause was the absence of one foundational access control. In a trial context, equivalent failures would compromise participant confidentiality, corrupt trial data integrity, and constitute reportable serious breaches under ISO 14155:2026. Required controls include risk-based access control, data minimization, encryption at rest and in transit, comprehensive audit logging, and a documented incident response procedure. In Taiwan, data collection, processing, and utilization must comply with the PDPA (46); IEC 81001-5-1 provides the applicable health software security lifecycle framework. For Taiwan-based trials, these controls should be implemented consistently with the PDPA and institutional information-security requirements, with inter-institutional or cross-border data access assessed according to the applicable legal and governance context.

### 4.5 Model drift and lifecycle change management

Unlike fixed-function clinical software, agentic AI is susceptible to performance degradation as patient populations, clinical workflows, or data infrastructure evolve—and this degradation may not be visible until audit or adverse outcome review. Subasri and colleagues analyzed EHR data from 143,049 inpatients across seven Toronto hospitals and found that significant data shifts—arising from changes in hospital type, patient demographics, admission source, and laboratory assay configurations—caused clinically meaningful model performance degradation; drift-triggered continual learning recovered performance with a *Δ*AUROC of 0.44 during the COVID-19 pandemic (47). The same mechanism applies when an agentic AI system crosses trial sites: differences in patient populations, device configurations, data formats, or clinical workflows constitute a change in operating environment that requires site-specific performance assessment before deployment. Trial governance must therefore pre-specify monitoring thresholds, recalibration triggers, validation requirements after material changes, and rollback procedures before AI deployment. PCCP (Predetermined Change Control Plans, PCCP)-type lifecycle change management, informed by FDA guidance and emerging international practice, provides a useful structural model. TFDA has also issued PCCP guidance for AI/ML-enabled medical device software; however, its applicability to agentic AI used as clinical-trial support infrastructure remains to be clarified.

## 5 Practical implementation pathways in Taiwan

### 5.1 TFDA-readable regulatory algorithms

TFDA regulatory pathways may be transformed into AI-readable decision workflows by encoding classification logic, evidence requirements, and CTP eligibility criteria as computable decision trees, enabling semi-automated regulatory intelligence and trial pathway selection. In Taiwan, the development of tools such as MEDPilot—which integrates large language models with retrieval-augmented generation to assist FDA pre-submission preparation—demonstrates that AI-assisted regulatory navigation is already technically feasible at the device-classification and predicate-identification level (22). Within the proposed framework,implementation must address two governance requirements. First, algorithmic bias (Domain 1): any AI system that performs eligibility determination or regulatory classification must be validated against locally representative datasets before deployment, with pre-specified subgroup performance thresholds documented in the trial protocol and predefined corrective actions if those thresholds are not met. Second, transparency and traceability (Domain 2): each regulatory classification decision must be accompanied by a traceable record of the TFDA guideline version referenced, the data inputs used, the classification logic applied, and the system's confidence level. Mandatory expert validation by a qualified regulatory professional remains a prerequisite before any AI-generated classification is acted upon.

### 5.2 Real-world data infrastructure and UDI integration

Taiwan possesses substantial real-world data assets—including NHIRD, hospital electronic medical records, disease registries, and continuous data streams from smart devices—that could strengthen the evidence base for device trials. Broader deployment requires IRB-approved data access agreements and governance infrastructure addressing the cybersecurity and privacy domain (Domain 4). All collection, processing, and utilization of clinical trial data by agentic AI must comply with Taiwan's Personal Data Protection Act (PDPA), including data minimization, purpose limitation, and restrictions on cross-border data transfer (45). Risk-based access control must be implemented at the level of individual agent functions: read access, write access, and decision-output permissions should be differentiated by agent role and data sensitivity, not granted uniformly. IEC 81001-5-1 provides the applicable health software security lifecycle framework. On the infrastructure side, a UDI-linked device registry—enabling linkage of device-specific data to NHIRD administrative records—is a priority investment for supporting AI-enabled PMCF and real-world evidence generation.

### 5.3 End-to-end agentic workflow architecture

The proposed end-to-end agentic workflow comprises specialised agents for protocol optimization, data mapping, recruitment matching, safety monitoring, and regulatory documentation generation. The accountability and human oversight requirements (Domain 3) define the operational boundaries of this architecture. Deploying agentic AI does not transfer legal or ethical responsibility from the principal investigator or sponsor; ISO 14155:2026 accountability obligations remain intact. A defined category of consequential actions must be reserved for documented human authorization before execution: protocol amendments to the CIP or statistical analysis plan; participant withdrawal decisions where AI monitoring flags safety signals or eligibility loss; and safety escalation involving classification and reporting of adverse device effects or serious adverse events. For each consequential action, the governance architecture requires the sequence: AI identifies or proposes → PI or delegate reviews the evidentiary basis → human authorises or overrides → authorization is documented with timestamp, responsible person, and rationale. Workflow architecture specifications should document which agent outputs require human authorization and at which frequency, pre-specified in the trial protocol and data management plan before AI deployment. The practical implementation of this workflow architecture in Taiwan requires careful attention to the clinical investigation infrastructure. Taiwan's major medical centres—including Chang Gung Memorial Hospital, National Taiwan University Hospital, and Taipei Veterans General Hospital—maintain mature electronic medical record systems and established clinical trial units, providing the institutional foundation for deploying agentic AI within device trials. However, the heterogeneity of EMR systems across institutions, variations in data coding practices, and differences in clinical workflow organization mean that an agentic system validated at one site cannot be assumed to perform equivalently at another without site-specific calibration. The framework's modular architecture—with discrete agents for protocol optimization, data mapping, recruitment, monitoring, and documentation—is designed to facilitate this site-specific adaptation by allowing individual agents to be reconfigured or retrained independently, without restructuring the entire workflow. This modularity also supports a phased deployment strategy in which individual agentic functions are introduced and validated sequentially, rather than deploying the complete five-agent system simultaneously.

### 5.4 Closed-loop PMCF and regulatory feedback

The continuous integration of trial execution data, PMCF evidence, adverse event reports, and registry-based surveillance supports adaptive redesign of device trials and dynamic accumulation of regulatory-grade evidence. Realising this potential requires the model drift and lifecycle change management governance described in Domain 5. For TFDA-regulated device trials, the closed-loop PMCF architecture must pre-specify before AI deployment: monitoring thresholds that trigger formal performance review; recalibration conditions and authorization requirements; re-validation requirements after material changes to the AI model, training data, or operating environment; and rollback procedures for reverting to a prior validated version if a new version fails performance requirements or introduces safety concerns. When the closed-loop system expands to additional sites, each new site should be treated as a potential change in operating environment requiring site-specific performance assessment before deployment, since differences in patient populations, device configurations, data formats, and clinical workflows may produce domain-specific performance disparities that aggregate metrics do not reveal. Taiwan's NHIRD offers a distinctive advantage for closed-loop PMCF: its near-universal population coverage enables longitudinal tracking of device performance outcomes at a scale difficult to achieve through investigator-initiated registries alone. Linking device-level data (via UDI) to NHIRD administrative records would create a foundation for continuous, population-level post-market evidence generation. The framework's Adaptive Feedback Layer (Layer 5) is designed to operationalize this linkage, using agentic AI to aggregate, analyze, and synthesize post-market data streams into actionable regulatory intelligence that feeds back to Layers 1 and 2. However, realising this vision requires not only technical infrastructure but also governance agreements between TFDA, the National Health Insurance Administration, and participating clinical institutions regarding data access, privacy safeguards, and the regulatory status of AI-generated post-market analyzes.

## 6 Actionable recommendations

Based on the preceding regulatory assessment, evidence synthesis, and governance analysis, we propose actionable recommendations organized by stakeholder group and implementation priority. Priority levels reflect estimated urgency and feasibility: high-priority actions address immediate regulatory and safety gaps; medium-priority actions strengthen infrastructure and standardization; lower-priority actions support longer-term ecosystem development.

### 6.1 For TFDA and regulatory authorities

[HIGH PRIORITY — 2026–2027] (a) Develop device-specific guidance for AI/ML and agentic AI systems used within clinical investigations, clarifying SaMD status determination and applicable lifecycle controls under the TFDA classification framework (13, 39). [HIGH PRIORITY — 2027–2028] (b) Establish a pilot regulatory sandbox or pre-submission consultation pathway for sponsors proposing to deploy agentic AI within TFDA-regulated device trials, enabling iterative dialogue before formal CTP submission. [MEDIUM PRIORITY — 2028–2029] (c) Clarify TFDA-specific requirements for AI lifecycle change management in the clinical-trial context, including the scope of permissible algorithm modifications during an ongoing investigation and corresponding re-validation obligations, drawing on the FDA's PCCP approach as an international reference.

### 6.2 For clinical investigators and sponsors

[HIGH PRIORITY — before trial initiation] (a) Before deploying any agentic AI component, formally assess its SaMD status under applicable TFDA and international frameworks, and document this assessment in the Clinical Investigation Plan. [HIGH PRIORITY — protocol design stage] (b) Pre-specify in the trial protocol which agent outputs constitute consequential actions requiring documented human authorization (at minimum: protocol amendments, participant withdrawal decisions, and safety escalations), and define the authorization workflow, responsible person, and audit-trail requirements. [MEDIUM PRIORITY — pre-deployment validation] (c) Validate AI components against locally representative datasets reflecting Taiwan's patient demographics, comorbidity profiles, and clinical workflows, with pre-specified subgroup performance thresholds and predefined corrective triggers. [MEDIUM PRIORITY — site expansion] (d) Treat expansion to a new investigation site as a change in operating environment, requiring site-specific performance assessment before deployment.

### 6.3 For AI developers and health IT

[HIGH PRIORITY — design phase] (a) Build agentic AI systems with modular, auditable architectures that maintain structured records of evidence sources, input–output provenance, tool-use logs, decision rationale, uncertainty indicators, and version history. [HIGH PRIORITY — deployment phase] (b) Implement risk-based access controls differentiated by agent function and data sensitivity, compliant with PDPA requirements and aligned with IEC 81001-5-1 security lifecycle principles. [MEDIUM PRIORITY — ongoing operations] (c) Pre-specify monitoring thresholds for model-drift detection, recalibration triggers, post-change validation requirements, and rollback procedures, following a PCCP-type change-management approach.

### 6.4 For infrastructure development

[HIGH PRIORITY — 2026–2028] (a) Prioritize the development of a UDI-linked device registry enabling linkage of device-specific data to NHIRD administrative records, as a foundation for AI-enabled post-market evidence generation. [MEDIUM PRIORITY — 2027–2029] (b) Support collaborative development, between regulatory authorities, clinical societies, and device manufacturers, of standardized and validated sensor-based endpoint definitions suitable for regulatory submissions. [MEDIUM PRIORITY — 2028–2030] (c) Invest in interoperable data infrastructure connecting hospital EMRs, disease registries, and smart-device data streams under PDPA-compliant governance frameworks.

## 7 Discussion

### 7.1 Bridging the translation gap

AI trial frameworks developed for pharmaceutical research do not port directly to devices, where engineering endpoints, operator-dependent variability, usability constraints, device-specific adverse-event classification, and device-specific safety considerations and lifecycle evidence obligations, including post-market evidence generation where applicable create a different operational landscape. The framework integrates TFDA classification logic, ISO 14155 compliance, and PDPA governance into a five-layer architecture (regulatory obligations per layer are detailed in Section 2.4). Published evidence—22.8% of FDA-cleared orthopaedic AI devices untested (37); high-risk implants often lacking comparative PROM data in European registries (38)—underscores the gap that AI-enabled PMCF automation could help address. The emergence of Taiwan-specific AI regulatory tools further reinforces this gap: systems like MEDPilot (22) demonstrate that AI can navigate FDA classification and predicate databases, but no equivalent tool addresses TFDA-specific device classification or CTP pathway determination, let alone the downstream trial-management functions covered by the framework's Layers 3–5. The IMDRF SaMD Clinical Evaluation framework provides an international benchmark for validating AI software, but its three-level evaluation model (valid clinical association, analytical validation, clinical validation) was designed for diagnostic or therapeutic SaMD, not for agentic systems that coordinate multiple trial functions. Adapting these evaluation concepts to the trial-support context is a regulatory development need that Taiwan could address through its existing TFDA–academia–industry consultation mechanisms.

### 7.2 Smart implant data and adaptive endpoints

Sensor-derived gait metrics and PROMs are weakly correlated but complementary; objective metrics may reveal recovery patterns PROMs miss. Future orthopaedic device trials may benefit from dual-endpoint designs, though clinical utility requires prospective validation and no validated minimum clinically important differences yet exist for sensor-derived endpoints. The Taiwan clinical environment is well positioned for this development: several major medical centres already have experience with sensor-enabled orthopaedic implants, and NHIRD's population-level administrative data provides a unique opportunity for linking device-level sensor data to long-term outcome records. However, realising this potential requires solving several practical challenges: standardization of sensor data formats across manufacturers, establishment of minimum clinically important difference thresholds for sensor-derived endpoints, and development of validated data-quality criteria for registry-based PMCF. The framework's Data Integration Layer (Layer 3) and Adaptive Feedback Layer (Layer 5) are designed to accommodate these requirements, but their operationalisation depends on infrastructure investments and multi-stakeholder collaboration that extend beyond any single trial.

### 7.3 Limitations

Six limitations apply, as follows. First, the framework is a conceptual design not evaluated through prospective implementation. Second, the illustrative cases do not validate the framework—weak Spearman's rho motivates but does not demonstrate effectiveness. Third, no AI-workflow study was designed for TFDA-regulated device trials, limiting transferability. Fourth, the regulatory layer encodes TFDA requirements, so FDA, EU MDR, or PMDA deployment requires adaptation. Fifth, cases are orthopaedic only, so applicability to cardiovascular, neurological, ophthalmic, and IVD trials is unverified. Sixth, the governance domains proposed in Section 4 are based on documented international cases, but their direct applicability to Taiwan's regulatory and healthcare context has not been empirically tested. Cultural, institutional, and legal differences between the jurisdictions in which the cited cases occurred and Taiwan's clinical investigation environment may affect both the severity and the optimal mitigation strategy for each governance risk. Additionally, the actionable recommendations in Section 6 propose specific timelines, but these are indicative rather than evidence-based; actual implementation pace will depend on TFDA regulatory priorities, available funding, institutional readiness, and the maturity of the AI systems proposed for deployment.

### 7.4 Future validation roadmap

We envision a staged validation pathway, contingent on funding, data access, ethics approval, and regulatory clearance. A first phase would seek expert-consensus assessment from independent clinical-investigation, regulatory, and AI professionals. A second phase would retrospectively apply the framework to completed device-trial documentation to identify gaps. A third phase would pilot selected agentic functions at a qualified investigation site, measuring protocol-deviation detection, processing time, human-override frequency, and regulatory acceptability. Until prospective data are available, the framework remains a conceptual proposal.

### 7.5 Conclusion

The framework's value ultimately depends not on its conceptual elegance but on whether it can survive contact with real clinical investigation environments. The staged validation pathway—expert assessment, retrospective testing, and prospective feasibility evaluation—is designed to test this incrementally. We encourage TFDA, clinical investigators, and AI developers to treat these recommendations not as prescriptions but as starting points for the iterative dialogue that responsible deployment of agentic AI in device trials will require.

We have outlined a five-layer agentic AI framework tailored to Taiwan's medical device clinical trial ecosystem. Its contribution lies not in a new algorithm but in an implementation architecture that ties agentic AI capabilities to the regulatory, evidentiary, and post-market realities specific to device development. Orthopaedic smart-implant examples illustrate how continuous device-derived data may complement conventional outcomes and how AI's role varies with regulatory evidence requirements—demonstrating applicability rather than validation. Governance runs through the architecture rather than sitting alongside it: bias monitoring, traceability, human oversight, cybersecurity, and model-drift controls are built into the operational workflow, and the investigator and sponsor keep full accountability for consequential decisions.

At this stage, the framework is a contextual proposal for Taiwan, not a validated system. The staged pathway—expert assessment, retrospective applicability testing, and prospective feasibility evaluation—provides a route from concept to evidence. Priority needs include interoperable UDI-linked real-world data infrastructure, standardized validation of device-derived endpoints, and regulatory clarity on AI lifecycle change management and SaMD governance. Whether the framework can serve device categories and jurisdictions beyond its current scope depends on validation studies we have yet to conduct.
